# Health Technology Assessment of Vaccines in Italy: History and Review of Applications

**DOI:** 10.3390/vaccines12101090

**Published:** 2024-09-24

**Authors:** Sara Boccalini, Rosalia Ragusa, Donatella Panatto, Giovanna Elisa Calabrò, Paolo Angelo Cortesi, Gabriele Giorgianni, Carlo Favaretti, Paolo Bonanni, Walter Ricciardi, Chiara de Waure

**Affiliations:** 1 Department of Health Sciences, University of Florence, 50134 Florence, Italy; paolo.bonanni@unifi.it; 2 HTA Committee, Azienda Ospedaliero Universitaria Policlinico “G. Rodolico-San Marco”, 95123 Catania, Italy; ragusar@unict.it; 3 Department of Health Sciences (DISSAL), University of Genoa, 16132 Genoa, Italy; panatto@unige.it; 4 Interuniversity Research Centre on Influenza and Other Transmissible Infections (CIRI-IT), 16132 Genoa, Italy; 5 Section of Hygiene, Department of Life Sciences and Public Health, Università Cattolica del Sacro Cuore, 00168 Rome, Italy; giovannaelisa.calabro@unicatt.it (G.E.C.); walter.ricciardi@unicatt.it (W.R.); 6 Research Centre on Public Health (CESP), University of Milano-Bicocca, 20132 Monza, Italy; paolo.cortesi@unimib.it; 7 UOS Coordinamento Operativo delle Attività Vaccinali Metropolitane e Provinciali–ASP 3 Catania, 95128 Catania, Italy; gabriele.giorgianni@aspct.it; 8 Centre on Leadership in Medicine, Università Cattolica del Sacro Cuore, 00168 Rome, Italy; carlo.favaretti@unicatt.it; 9 Department of Medicine and Surgery, University of Perugia, 06123 Perugia, Italy; chiara.dewaure@unipg.it

**Keywords:** HTA, vaccination, immunization, recommendation, NITAG, decision-making process, market access, stakeholder

## Abstract

**Background/Objectives**. Many vaccines have been developed in recent decades, and many more will be available in the future. When new safe and effective vaccines are available, decision-makers must extensively assess them before including them in the national immunization plan and issuing recommendations. The Health Technology Assessment (HTA) could be an objective, transparent, and comprehensive approach to guiding the decision-making process for the use of vaccines. **Objectives and Methods**. The aim of this study was to review the indications for HTA use contained in Italian institutional documents on vaccination, namely the National Immunization Plans (NIPs) and available full Italian HTA reports on vaccines, assessing their availability at the time of national recommendations’ introductions. **Results**. HTA has been recognised as an eligible approach to deciding upon the introduction of vaccines through the NIPs of 2012–2014 and 2017–2019, and the last NIP, of 2023–2025, highlights the lack of funding dedicated to the production of independent HTA reports that can be used for issuing recommendations. In 2007–2023, twenty full HTA reports on vaccines were published in Italy: eight reports on influenza vaccines, five on Human Papilloma Virus (HPV), three each on meningococcal and pneumococcal vaccines, and one on rotavirus vaccine. HTA was applied with different purposes, namely the evaluation of new vaccines or their re-assessment, but it was not always timely with respect to both the marketing authorisation and the issuing of national recommendations for use. **Conclusions**. As HTA can be considered the best tool to disentangle the overall value of vaccines, it would be desirable for it to be used more and more to provide the evidence for efficient resource use. This calls for action to improve the transfer of HTA results to decision-makers, to try to fill the gap between research and decision and foster evidence-based recommendations.

## 1. Introduction

Vaccines are the health intervention with the most effect on mortality reduction and population growth, after safe water [1,2,3,4,5,6,7,8,9]. Many vaccines have developed in recent decades, and many more will be available in the future [10,11,12,13,14,15]. When new safe and effective vaccines are available, decision-makers must extensively assess them before issuing recommendations on their inclusion in national or regional immunization plans.

There are four basic values considered in the assessment of vaccines and in the subsequent decision-making process behind the issuing of recommendations. These core values are the incidence of the vaccine-preventable disease, its lethality, its severity, and the cost-effectiveness of vaccination [16,17]. However, other issues, such as the impact on non-health outcomes, societal impact, and pragmatic issues should be considered in the decision-making process, as suggested by the World Health Organization [18,19,20].

An objective, transparent, and comprehensive approach that is useful for addressing all these issues and supports the National Immunization Advisory Group (NITAG) is the Health Technology Assessment (HTA). HTA is a systematic and multidisciplinary evaluation of health technologies that addresses the potential direct and indirect consequences of their use. It aims to determine the value of a health technology and to inform how it can be used in the best way in health systems. HTA relies on the collection and synthesis of evidence regarding the medical–clinical, social, organisational, economic, ethical, and legal implications of a health technology, evaluating multiple dimensions such as efficacy/effectiveness, safety, cost, and social–organisational impact. Therefore, HTA can be used to support the decision-making process in health care by providing evidence about given technologies [21,22,23,24,25,26].

HTA has reached a juncture where relevant and impactful changes for European citizens are expected. The HTA Regulation 2021/2282 [27] will apply as of January 2025 and will call for a harmonized HTA among member states to improve access to health technologies. Vaccines will adhere to this HTA Regulation starting from 2030. Actually, a deep heterogeneity in the assessment of vaccines is emerging among NITAGs [28,29]. In Italy, the NITAG was established only recently and did not immediately reach the target of becoming a reference point for Italian vaccination policies [30]. Nevertheless, a long-lasting history regarding HTAs of vaccines is in place in Italy, with the first HTA report being published 10 years ahead of the establishment of the NITAG. The inclusion of a vaccine in the Italian National Immunization Plan (NIP) is decided by the Ministry of Health, as in other European countries, representing a critical step for Italy due to the need for unanimous regional consensus [31]. To date, the interlinkages between HTAs and vaccination recommendations issued at a national level have not been extensively addressed in the literature. Several works have focused on aspects used by NITAGs or HTA bodies to issue recommendations on specific vaccines, providing a more methodological view of the topic [32,33,34]. In this light, studies in countries from which information on HTAs of health technologies other than medicines is less common are appropriate [35]. As a matter of fact, HTAs on vaccines are uncommon relative to clinical interventions or other public health activities [36].

The aim of this study was to evaluate the use of HTA in the landscape of vaccines in Italy though an overview of the official indications provided by vaccine-relevant Italian institutional documents and of the available full Italian HTA reports on vaccines, assessing their availability at the time of national recommendations’ introductions.

## 2. Materials and Methods

The study was performed in two steps. In the first phase we retrieved official indications for the use of HTA in the vaccine-related decision-making process. Therefore, we collected vaccine-relevant institutional documents, namely Italian NIPs, and searched for “HTA” or “Health Technology Assessment” or “valutazione delle tecnologie sanitarie” to extract any information on the use of HTA. In the presentation of the results, we described whether HTA was reported as an evaluation approach for the assessment of vaccines, and we reported any change in this topic that occurred over time. NIPs were chosen because they are national programmes addressing vaccine-preventable disease control that are issued by the Italian Ministry of Health, which is the main institution responsible for public health at the national level. All the available NIPs from 2000 to June 2024 were considered for this study. The year 2000 was chosen as the 1999 reform of the Italian National Health Service reaffirmed the central role of the government in setting the national health policy agenda [37]. In the second phase we systematically searched and collected full HTA reports on vaccines published in Italy from 2000 to the present. Particularly, a hand search was performed on the websites of two journals (*Italian Journal of Public Health* and *Journal of Preventive Medicine and Hygiene*) using a combination of keywords including “HTA”, “Health Technology Assessment”, “valutazione delle tecnologie sanitarie”, “vaccino”, “vaccinazione” [38,39]. These two journals were selected as they are committed to publishing full HTA reports on vaccines in Italy. The hand search of these two journals was also considered a reasonable approach because national HTA reports are published in Italian, and the two journals allow the publication of single-issue supplements in Italian. The hand search was performed by one researcher and the results were validated through consultation with all of the working group. HTA reports were considered eligible if they were full reports that described the characteristics and current use of the technology, its safety and efficacy, and its cost-effectiveness, and if they provided information on its costs/financial impact and organisational aspects [40]. The collected HTA reports were grouped together according to the vaccine-preventable disease. The examined vaccine types and target population/vaccination strategy were evaluated following a chronological order. In addition, the year of publication of the reports was related with the year of vaccine marketing authorization issued by the Italian Medicines Agency (Agenzia Italiana del Farmaco—AIFA) and the national recommendation included in the NIPs.

## 3. Results

### 3.1. Indications for Using HTA Based on Institutional Documents in Italy

In Italy four NIPs were issued from 2000 to the present: NIP 2005–2007 [41], NIP 2012–2014 [42], NIP 2017–2019 [43], and the current NIP, NIP 2023–2025 [44]. The NIP of 2012–2014 is the first institutional document on vaccinations where HTA is cited as an evaluation approach for the inclusion of vaccines in the NIP. As a matter of fact, this plan highlights the increasing number of vaccines over time and, consequently, the need to make rational use of the limited resources available to maximise the population’s health. To do this, “clear, robust and shared criteria” to support decision-making processes regarding the introduction of vaccines are needed. The HTA was, therefore, recognised as the approach that best meets these evaluation needs [42].

HTA has been confirmed as the most correct and transparent approach to supporting health decision-making by the NIP of 2017–2019. In this document it is also stressed that HTA is important and necessary because vaccinations are a public health intervention carried out on healthy people with intangible benefits that are not always adequately perceived (absence of disease and reductions in health care as well as the direct and indirect costs of the disease). In the assessment, therefore, beyond the costs of vaccines and vaccination, the short-, medium, and long-term benefits should be considered [43].

The current NIP of 2023–2025 specifies that the availability of new vaccines or new indications for existing and used vaccines represents an additional opportunity for the protection of the individual and also of the community. However, the introduction of new vaccines and the implementation of new vaccination strategies may be problematic due to the increased financial commitment (purchase of new vaccines or additional doses of existing vaccines, staff training, communication and dispersing information to the population, etc.) and the impact on the organisational system. The decision-making process leading to the introduction or implementation of a vaccine must, therefore, weigh up many aspects. These aspects may slow down or hinder the inclusion of vaccines in the national recommendations [44].

In addition, the NIP of 2023–2025 highlights the lack of a standardized decision-making process for the inclusion of vaccines in national recommendations and of funding dedicated to the production of independent cost-effectiveness analyses and HTA reports that can be used to issue recommendations by the NITAG and the Ministry of Health [44].

### 3.2. HTA Reports on Vaccines Published in Italy

The first full HTA report on a vaccine was published in Italy in 2007 [45]. In the time period considered, twenty full HTA reports on vaccines were published either in the *Italian Journal of Public Health* or in the *Journal of Preventive Medicine and Hygiene*: eight (40%) reports referred to influenza vaccines, five (25%) to HPV, three each (15%) to meningococcal and pneumococcal vaccines, and one to the rotavirus vaccine (5%). In Table 1 the full HTA reports are reported according to the type of vaccine [45,46,47,48,49,50,51,52,53,54,55,56,57,58,59,60,61,62,63,64].

In Italy, the HTA approach was first applied to assess the new HPV vaccines. As a matter of fact, the tetravalent HPV vaccine (HPV4) was authorized by the AIFA in 2006 [65] while the bivalent HPV vaccine (HPV2) was authorized in 2007 [66]. The HTA report on HPV2 was published in 2007 [45], while the one on HPV4 was published in 2009 [46]. These reports aimed to assess the use of the new and expensive vaccines against cervical cancer. The national HPV vaccination campaign targeting females in their twelfth year of age started in 2007/2008 based on the agreement between the Ministry of Health and the Italian regions and autonomous provinces (Intesa Stato-Regioni del 20 dicembre 2007), and the recommendation on HPV vaccination for female adolescents was then included in the NIP of 2012–2014 [42]. In 2014 the HTA approach was applied to re-assess the HPV vaccination 5 years after its introduction and to evaluate the two-dose schedule [47]. In 2015 the new 9-valent HPV vaccine was authorized in Italy [67] and in 2017 this new vaccine was assessed in an HTA report [48]. In 2019 an HTA report was performed to evaluate the use of the HPV9 vaccine in women treated for HPV-related diseases based on the availability of new evidence on the efficacy of vaccination in this risk group [49]. Nevertheless, this strategy was included only in the NIP of 2023–2025, even though it should be taken into consideration that in 2020 an official guideline on post-treatment vaccination was published [68].

The pneumococcal vaccination was another instance where HTA was applied to assess both new vaccines and their new indications for use in other target groups. The 13-valent conjugated pneumococcal vaccine (PCV13) was authorized by the AIFA for use in the paediatric population in December 2009 [69] and the first HTA report was published in 2010 [50]. Subsequently, based on new evidence, the vaccine was also approved for use in the adult population and a new HTA was published in 2013 [51]. Another conjugate vaccine, namely the 15-valent pneumococcal vaccine (PCV15), was authorized for use in people aged 18 years or older in 2021, and then approved for use in the paediatric population in 2022 [70]. In regard to this vaccine, an HTA report focused on the paediatric population was available one year later, after the marketing authorization [52]. Eventually, the 20-valent pneumococcal vaccine (PCV20) received authorization for use in people aged 18 years or older in 2022, with extension to the paediatric population in the last year [71], but no HTA reports are available yet. In respect to the preferential use of one vaccine over the other, it should be observed that only in the NIP of 2017–2019 we found a reference to administer the vaccine with the greater protection against circulant pneumococcal serotypes in the paediatric population [43]. The last NIP did not specify the type of conjugate vaccine that should be preferably used either in paediatric or in adult and elderly populations [44].

HTA in Italy has a broader application in the field of influenza vaccines. Five HTA reports [53,54,56,58,59] have been performed on different influenza vaccines (quadrivalent egg-based vaccine, adjuvanted vaccine, cellular-based vaccine, live attenuated vaccine, high-dose vaccine). Two reports [57,60] were reassessments of vaccines already evaluated that were conducted based on new indications/formulations. Lastly, a report [55] assessed the introduction of a paediatric vaccination strategy against influenza. All these reports were published from 2015 to 2023. The publication years of the HTA reports are close to the first influenza season in which each vaccine or its new indication were available, except for the adjuvanted trivalent vaccines, which have been used since 1997/1998 and were evaluated through HTA only in 2017 (Table 2).

Rotavirus vaccines were authorized by the AIFA in 2006 [72,73]; however, in this case, the HTA of the live attenuated vaccine was performed only in 2014 [61] and it took about ten years from marketing authorization to the introduction of rotavirus vaccination into the NIP of 2017–2019 [43] (even though many Italian regions had already adopted rotavirus vaccination at that time).

Concerning meningococcal vaccines, the quadrivalent meningococcal B vaccine for newborns was authorized in 2013 [74] and in the same year a specific HTA report was already available [62]. The recommendation for this vaccine was then included in the NIP of 2017–2019 [43], even though some Italian regions had already started to administer it at that time. The bivalent meningococcal B vaccine for adolescents had a different market access pathway: it had marketing authorization in 2017 [75] and an HTA was available in 2019 [63], but to date, there is still not a national recommendation to use it, although, based on the regional autonomy, some regions have started providing the vaccination free of charge. Concerning meningococcal C immunization, this vaccination has been recommended at 13–15 months of life and in adolescents from the 2012–2014 NIP onwards [42]. According to the increasing evidence of protection from meningococcal vaccination waning over time [76,77], and based on the availability of a quadrivalent meningococcal vaccine, a four-cohort strategy (immunization at 13–15 months, 6 years, 11 years, and 19 years of age) with a quadrivalent meningococcal vaccine was assessed in 2021 [64], but this multiple preventive intervention is not included in current recommendations.

## 4. Discussion

The aim of this study was to make an overview of the indications for the use of HTA in the vaccine-related decision-making process, as well as its actual application in Italy. The findings of our work are relevant to understanding how far the evidence supports the decisions on vaccination at the Italian level. The results are also of interest considering the growing attention to evaluating the functionality, but also the output, of NITAGs worldwide [78]. The results of the work highlight that the HTA approach has been recognized as an invaluable approach to guiding the vaccine-related decision-making process for more than 10 years in Italy. Nevertheless, the actual use of HTA in decision-making shows room for improvement. This could also be due to the history of the Italian NITAG that was established in 2017, dissolved, and then established again in its current composition in 2021 [30]. As a matter of fact, HTA reports on vaccines published in Italy were developed by academic research groups with companies’ unconditional grants, and this could have prevented their timely consideration in the development of recommendations [30]

Delving into specific diseases, we observed that, not surprisingly, HTA was first applied to HPV vaccines, which are very expensive preventive interventions with a great potential to control cancers. In respect to HPV vaccines, HTA was also adopted to reassess the impact of these vaccinations some years after their introduction and in light of new indications. These reassessment exercises are quite relevant to monitoring vaccination policies and for continuous quality improvement. A reassessment exercise was also observed with respect to pneumococcal conjugate vaccines, namely PCV13, based on new indications. As far as pneumococcal vaccines are concerned, one of the last new products, PCV15, was assessed through HTA, although national recommendations were not still impacted by the results of the report. The available HTA reports also belatedly or partially impacted NIPs with respect to rotavirus and meningococcal vaccinations. As a matter of fact, the HTA report on the rotavirus vaccine was performed many years after the marketing authorization and the immunization was included in the NIP. This delay in the introduction of rotavirus vaccination into the NIP could be also attributable to the low perception of the health problem and of its societal impact [79,80,81,82,83,84]. For meningococcal vaccines, the HTA reports are relevant to fully understanding the real impact of infection and immunization in Italy. In fact, in this case, the collection of scientific evidence highlights that, even if the infection has a very low incidence [85,86,87,88,89,90,91], the health impact (complications, short- and long-term sequelae, and deaths) is very significant [62,63,64,92,93,94,95,96,97]. This aspect is relevant in the decision-making process, even though it is not always sufficient to include the different vaccines and vaccination strategies in the national recommendations, probably due to economic issues.

Our findings show that, in Italy, HTA has been applied widely for influenza vaccination. This is related to the many types of vaccines available in recent years, each with specific characteristics. The different HTA reports have made it possible to understand that influenza vaccines are not similar; therefore, they must be administered according to appropriateness criteria [98]. All the HTA reports show that influenza vaccination, with any vaccine, is essential for avoiding complications, hospitalizations, and deaths, but that vaccination with the most suitable product for each type of subject could optimize health outcomes at a very modest additional cost. This kind of information is relevant when pursuing a value-based approach to vaccination and when issuing specific recommendations [99].

Overall, our findings suggest that in Italy decision-makers have not been using HTA to its full potential and this has also been reported in the NIP of 2017–2019 [43].

Potential reasons behind the non-use of HTA reports in Italy could be that they are not delivered by an independent national organization and that there is not a comprehensive and targeted dissemination strategy for their results. As a matter of fact, while in the NIPs of 2012–2014 [42] and 2017–2019 [43] HTA is recognised as the primary assessment approach for the introduction of new vaccines, the current NIP of 2023–2025 [44] highlights the gap in funding dedicated to the production of independent HTAs that can be used for developing vaccination recommendations by the NITAG and the Ministry of Health. Nonetheless, in order to make the best use of HTA, the NITAG, as well as the Ministry of Health and all the involved stakeholders, should be adequately trained [100]. This could also lay the foundation for a stronger collaboration among relevant bodies and entities. Although international efforts already exist in this respect, parallel activities should be carried out at national level [78].

In fact, the importance of HTA in assessing vaccines is unquestionable and supported by the WHO guidelines on the introduction of new vaccines [19]. Furthermore, the European Regulation (EU) 2021/2282 on HTA [27] underlines the importance of HTA as a tool to ensure the timely introduction and use of new technologies, with a view to enhancing sustainability and the promotion of innovation. Prevention measures, including vaccines, are among the health technologies in scope of the regulation. For the clinical domains (the health problem, the current health technology, the technical characteristics of the health technology being evaluated, its safety and relative clinical effectiveness), joint and shared evaluation, namely the Joint Clinical Assessment (JCA), will be expected among European Member States. The latter should give due consideration to JCA, although complementary analyses could also be carried out at a national level. Anyway, the final decision to use health technologies will be entrusted to single Member States that are responsible for defining their health policy and the organisation and provision of health services and medical care. Therefore, non-clinical domains (the cost and economic assessment of health technology and its ethical, organisational, social, and legal aspects) will continue to be assessed at a national level because they are strongly dependent on the national context [27].

HTA will indeed remain a cornerstone of the vaccine-related decision-making process, which is currently complex, lengthy, and heterogeneous across European countries because of their different roles, decision-making criteria, and transparency of NITAGs and HTA bodies [28,31].

The findings of our work allow an overall view of the current maturity level of HTA for vaccines in Italy, in terms of both its conduction and applications.

A limitation of the study that should be considered is that in Italy the NIP is not updated on a regular basis. This can explain the late introduction of some vaccines with respect to the available HTA reports. In this context, some Italian regions also introduce vaccines into their regional immunization plan before the national recommendation, but our study did not capture this aspect. In addition, we only searched full HTA reports, and it should be considered that sometimes decisions are based only on partial evaluations, mostly economic assessment. The considered HTA reports did not issue formal recommendations on the use of specific vaccines, but synthetized the available evidence for different domains. For this reason, we did not include the results of each report, also because the objective was not to determine how the final decision was achieved. The latter is based on the appraisal of the HTA reports that could benefit from deliberative approaches too. Furthermore, in Italy, HTA reports on COVID-19 vaccination were not performed, even though immunization was introduced quickly, as soon as vaccines were available. Another limitation is represented by the choice to conduct the search for information about the use of HTA in the vaccine-related decision-making process using only national planning documents, namely the NIPs. In any case, the purpose of the study was to describe how HTA is considered within the field of national health planning, although it should be kept in mind that, because of local autonomy, regions can develop different vaccination strategies. Nonetheless, the NIP is the common base for the organization of vaccination strategies in all Italian regions. Another limitation is due to the fact that the study relies on a hand search performed on selected national journals that are well known in terms of the provision of such evidence. Eventually, no formal indicators were considered to address the impact of HTA [101,102,103,104,105,106], but this was reasoned by the fact that the work did not have the aim to monitor and evaluate the implementation of vaccination strategies [107]. Anyway, this topic could also merit further investigation with respect to the potential role of HTA in impacting several policy indicators.

The main strength of this study is that it provides an overall evaluation of the use of HTA in the landscape of vaccines that is currently missing in Italy. The findings highlight some critical issues in the application of this approach and can be useful for the future implementation of HTA in the issuing of Italian recommendations for vaccinations.

## 5. Conclusions

Our results highlight that HTA production in Italy is wide-ranging and continuous and can provide relevant information for the use of new vaccines, but can also be used to reassess already available and used ones. This evidence could respond to the evaluation needs emphasized in the national vaccine-relevant planning documents. Future research should elucidate how to conduct HTAs that fit the purpose of achieving quality and sustainable vaccination programs and provide timely support for vaccine-related decision-making, also considering the improvement of the transfer of HTA results to decision-makers and all relevant stakeholders.

## Figures and Tables

**Table 1:** HTA reports on vaccination published in Italy in the period 2007–2023.

Year	HTA Report	Vaccine	Target Population/Vaccination Strategy	Reference
HPV vaccines				
2007	Health Technology Assessment of anti-HPV vaccination	HPV2	Female adolescents (12 years old)	[45]
2009	HTA report of the quadrivalent anti-HPV vaccine Gardasil	HPV4	Female adolescents (12 years old)	[46]
2014	Revaluation of anti-HPV vaccination 5 years after its introduction. HTA 2.0	HPV	Female adolescents (12 years old)/two-dose versus three-dose schedule	[47]
2017	The anti-HPV 9-Valent vaccine: Health Technology Assessment (HTA) report	HPV9	Adolescents (12 years old)	[48]
2019	In-depth report and evaluation, using HTA (Health Technology Assessment) methodology, of anti-HPV vaccination in women treated for HPV-related injuries	HPV	Women treated for HPV-related injuries	[49]
**Pneumococcal conjugate vaccines**				
2010	Report of Health Technology Assessment of anti-pneumococcal vaccination with Prevenar 13	PCV13	Paediatric population	[50]
2013	The 13-valent pneumococcal vaccine for the prevention of adult *S. pneumoniae* infections: an evaluation of HTA	PCV13	Older population	[51]
2023	The new 15-valent antipneumococcic conjugate vaccine for the prevention of *S. pneumoniae* infections in children: an assessment of HTA	PCV15	Paediatric population	[52]
**Influenza vaccines**				
2015	Health Technology Assessment of the FLU-QIV quadrivalent influenza vaccine (Fluarix Tetra)	Flu QIV	General population/quadrivalent vaccine versus trivalent vaccine	[53]
2017	Health Technology Assessment (HTA) of the adjuvant influenza vaccine in the elderly Italian population	Flu aTIV	Older population	[54]
2018	Universal vaccination of children against influenza with the vaccine Vaxigrip Tetra in Italy	Flu QIV paediatric population	Paediatric vaccination	[55]
2019	Health Technology Assessment (HTA) of the Flucelvax Tetra cell culture quadrivalent influenza vaccine	Flu ccQIV	General population	[56]
2021	Health Technology Assessment (HTA) of the adjuvant quadrivalent influenza vaccine: Fluad Tetra	Flu aQIV	Older population	[57]
2021	HTA report of the EFLUELDA high-dose quadrivalent vaccine (QIV-HD) for the prevention of seasonal influenza and its complications in the over-65 population	Flu hdQIV	Older population	[58]
2021	Health Technology Assessment (HTA) of the introduction of flu vaccination for the Italian youth population with the vaccine Fluenz Tetra	Flu LAIV	Paediatric population	[59]
2023	The Health Technology Assessment as a value-based tool for evaluating healthcare technologies Reassessment of quadrivalent cell culture influenza vaccine: Flucelvax Tetra 2.0	Flu ccQIV	General population	[60]
**Rotavirus vaccine**				
2014	Health Technology Assessment of Rotavirus vaccination with the Rotarix vaccine	Rotavirus	Infant population	[61]
**Meningococcal vaccines**				
2013	Health Technology Assessment of vaccination against Meningococcus B	Quadrivalent MenB	Infant population	[62]
2019	Health Technology Assessment of meningococcal B vaccine (Trumenba^®^) in adolescents in Italy	Bivalent MenB	Adolescents	[63]
2021	Health Technology Assessment (HTA) on the introduction of additional cohorts for vaccination against meningococcus with quadrivalent conjugated vaccines in Italy	MenACWY	2-cohort strategy (subjects of 13–15 months and 11 years) versus 4-cohort strategy (subjects of 13–15 months, 6 years, 11 years, and 19 years of age)	[64]

Note: HPV2: bivalent HPV vaccine; HPV4: quadrivalent HPV vaccine; HPV: HPV vaccine; HPV9: 9-valent HPV vaccine; PCV13: 13-valent conjugate vaccine; PCV15: 15-valent conjugate vaccine; Flu QIV: quadrivalent inactivated influenza vaccine; Flu aTIV: trivalent inactivated adjuvated influenza vaccine; Flu ccQIV: quadrivalent inactivated cellular culture influenza vaccine; Flu aQIV: quadrivalent inactivated adjuvated influenza vaccine; Flu hdQIV: quadrivalent inactivated high dose influenza vaccine; Flu LAIV: live attenuated influenza vaccine; Rotavirus: rotavirus vaccine; Quadrivalent MenB: quadrivalent meningococcal B vaccine; Bivalent MenB: bivalent meningococcal B vaccine; MenACWY: meningococcal ACWY vaccine.

**Table 2:** HTA reports on influenza vaccination: year of publication, influenza season of vaccine availability, and national recommendation.

HTA Report	Vaccine	Year of Report Publication	First Influenza Season of Vaccine Availability and National Recommendation ^1^	Vaccination Program/Extension of the Indications of Use
Assessment of the health technology of the FLU-QIV quadrivalent influenza vaccine (Fluarix Tetra) [53]	Flu QIV	2015	2014–2015	
Health Technology Assessment (HTA) of the adjuvant influenza vaccine in the elderly Italian population [54]	Flu TIVa	2017	1997–1998	
Universal vaccination of children against influenza with the vaccine Vaxigrip Tetra in Italy [55]	Flu QIV paediatric population	2018	-	Pediatric vaccination program from 2020–2021 influenza season
Health Technology Assessment (HTA) of the Flucelvax Tetra cell culture quadrivalent influenza vaccine [56]	Flu QIVcc	2019	2019–2020	
Health Technology Assessment (HTA) of the adjuvant quadrivalent influenza vaccine: Fluad Tetra [57]	Flu QIVa	2021	2021–2022	
HTA report of the EFLUELDA high-dose quadrivalent vaccine (QIV-HD) for the prevention of seasonal influenza and its complications in the over-65 population [58]	Flu QIVhd	2021	2020–2021	
Health Technology Assessment (HTA) of the introduction of flu vaccination for the Italian youth population with the vaccine Fluenz Tetra [59]	Flu LAIV	2021	2021–2022	
The Health Technology Assessment as a value-based tool for evaluating healthcare technologies Reassessment of quadrivalent cell culture influenza vaccine: Flucelvax Tetra 2.0 [60]	Flu QIVcc	2023	2021–2022	2021: extension of the indications of use ≥2 years of age

^1^ Annual Ministerial Circular for the prevention and the control of seasonal influenza.

## Data Availability

No new data were created or analyzed in this study. Data sharing is not applicable to this article.
